# 63 km BOFDA for Temperature and Strain Monitoring

**DOI:** 10.3390/s18051600

**Published:** 2018-05-17

**Authors:** Thomas Kapa, Andy Schreier, Katerina Krebber

**Affiliations:** Bundesanstalt für Materialforschung und -prüfung, Unter den Eichen 44-46, 12203 Berlin, Germany; andy.schreier@bam.de (A.S.); katerina.krebber@bam.de (K.K.)

**Keywords:** distributed sensing, stimulated Brillouin scattering, fiber optics sensors

## Abstract

We demonstrate (and are the first to do so) 63 km Brillouin Optical Frequency-Domain Analysis (BOFDA) for temperature and strain monitoring using a 100 km fiber loop. The use of BOFDA for long-range applications can be considered a novel approach, as previous investigations focused on the utilization of Brillouin Optical Time-Domain Reflectometry and Analysis (BOTDR and BOTDA, respectively). At 51.7 km, a 100 m hotspot (37 ${}^{\circ }$C) was detected without using distributed Raman amplification or image processing.

## 1. Introduction

Brillouin scattering is used in a wide range of applications, such as condition monitoring of pipelines [1], dikes [2], river embankments [3], and high-voltage cables [4], employing a multitude of measurement methods [5,6]. Two common measurement methods are Brillouin Optical Time-Domain Analysis (BOTDA) and Brillouin Optical Frequency-Domain Analysis (BOFDA). We focus on developing BOFDA, in particular for use for the above-mentioned applications.

BOTDA has already overcome enormous measurement lengths up to 120 km with a spatial resolution of 5 m, or measurement lengths of 50 km with a spatial resolution of 1 m, with a measurement time in the minute range [7,8,9]. In comparison, about 11 km, 5 km, and 3 km results of BOFDA have been reported [10,11,12]. The further extension of the measurement range for BOFDA has been a nearly untreated topic in the last decade. The main reason is probably the increase in measuring time with increasing sensor length due to the necessary narrow-band filtering of the measured signals. To circumvent this disadvantage of long measuring times, the slope-assisted approach [13,14] is promising for decreasing measuring time. A second reason is that, for very long fibers, the interaction between stationary lightwaves is supposed to lead to a strong depletion of the pump, resulting in an unsatisfactory SNR in the farthest region of the fiber.

In this paper, we demonstrate (and are the first to do so) 63 km BOFDA for temperature and strain monitoring using a 100 km fiber loop. The sensing part of the fiber is 63 km. To extend the measurement length up to this 63 km, we employed a method of spatially multiplexed frequency-shifted fibers [15]. In this method, optical fibers with different Brillouin frequency shifts (BFSs) are used.

## 2. Experimental Setup

**Figure 1:**
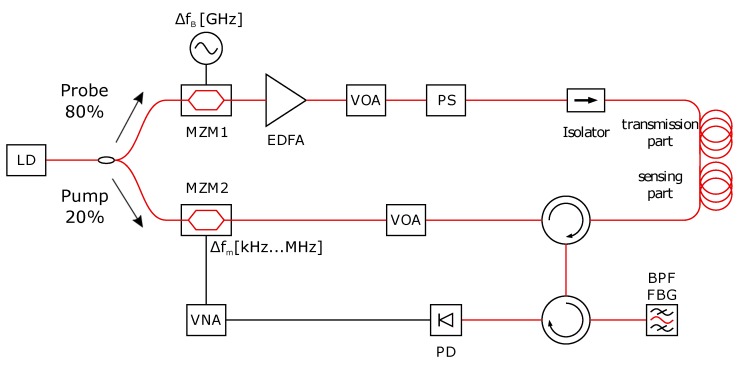
BOFDA sensor setup. LD: laser diode; MZM: Mach–Zehnder modulator; EDFA: erbium-doped fiber amplifier; VOA: variable optical attenuator; PS: polarization scrambler; SMF: single mode fiber (for detailed configuration, see Figure 3); BPF FBG: bandpass filter fiber Bragg grating; PD: photodiode; VNA: vector network analyzer.

The proposed sensor setup is shown in Figure 1. Our BOFDA setup required the use of only one laser [12]. The laser diode (LD) was a distributed feedback laser (DFB laser, EM4 EM600 2008-11-22 REV1) emitting at 1550 nm with a narrow linewidth of 300 kHz and a maximum output power of 105 mW. A high power laser was used for two reasons. Firstly, the power of the light was split into two paths of 20 and 80% of the power for the pump and the probe in the lower and upper branches of the setup, respectively. Secondly, the light in the probe branch was modulated by the Mach–Zehnder modulator (MZM) MZM1 operating on the minimum of the transfer curve to suppress the carrier (dual sideband with suppressed carrier, DSB-SC, [16]). These two facts significantly reduced the power in the probe branch. These are the reasons why an erbium-doped fiber amplifier (EDFA) was used in the probe branch.

The setup includes two MZMs. MZM2 was modulated on the quadrature operating point to operate in the linear region of the transmission curve for the measurement of the complex transfer function H($\Delta f_{m}$). The transfer function originates from the transfer of the modulated pump power into a power modulation of the probe by stimulated Brillouin scattering (SBS) measured by a photo-detector. MZM2 was modulated by a vector network analyzer (VNA, Keysight E5061B).

MZM1 is modulated around the BFS by a signal generator. A narrow linewidth of the laser is required to minimize the noise of the Brillouin gain [17,18]. The BGS was scanned with a 4 MHz frequency step and a span of 96 MHz. The MZM1 generated an upper and a lower side band but only the lower sideband was used to amplify the Brillouin scattering. The output signal of MZM1 was amplified by an EDFA with automatic gain control and was fed into a variable optical attenuator (VOA) to regulate the power. In a standard single mode optical fiber, the polarization state of propagating laser light varies randomly. This is also the case for the pump and the probe light in our setup. Our setup includes an additional polarization scrambler with a polarization variation frequency of 700 kHz to completely ensure an interaction between the counter-propagating pump and probe. In this way, the refracted signal level increases, and insensitive fiber parts due to orthogonal pump and probe waves were avoided [19,20]. An isolator, placed after the PS, protected the previous components from the counter-propagating pump wave and the signal reached the fiber loop.

The modulation frequency of the pump light by the VNA have to be in the range of 1 kHz–4 MHz. After inverse fast Fourier transformation (iFFT) of H($\Delta f_{m}$), the frequency step $\Delta f_{m}$ is inversely proportional to the measurement length, while the upper modulation frequency is inversely proportional to the spatial resolution. The lower modulation frequency was adjusted to 1 kHz for a fiber length of 100 km, and the upper frequency was adjusted to 4 MHz to obtain a spatial resolution of 26 m (Equation (1) [21]):

(1) $$\Delta z=\frac{c}{2n}\frac{1}{f_{m,max}-f_{m,min}}.$$

Here, *n* is the refractive index of the optical fiber, *c* is the speed of light in vacuum, and $f_{m,max}$ and $f_{m,min}$ are the upper and lower modulation frequency, respectively, as modulated by the VNA.

For the iFFT, a Kaiser window [22] was used with a Beta-Factor of 5.

The lower cutoff frequency of the detector, the modulator, and the VNA had to correspond to $\Delta f_{m}$. All these components had to be able to measure this lower cutoff frequency component. An $L_{max}$ of 100 km corresponds to a lower cutoff frequency of 1 kHz (Equation (2), corresponding to [21], and from OFDR [23,24]):

(2) $$\Delta f_{m}=\frac{c}{2n}\frac{1}{L_{max}}.$$

An important parameter of the VNA is the intermediate frequency (IF) bandwidth. This parameter determines the filtering bandwidth around each data point of the complex transfer function and should not exceed the lower frequency component, in our case 1 kHz. For our measurement, we chose 500 Hz. The step size $\Delta f_{m}$ of the modulation of VNA is also determined by the lower frequency and was therefore set to 1 kHz. The H($\Delta f_{m}$) was measured by the VNA at 4000 frequency steps in order to realize a measurement length of 100 km and a spatial resolution of 26 m results. This, in turn, results in a measuring time of 8 s for an IF bandwidth of 500 Hz for one measurement without averaging. We chose 250 averages and 24 steps of $\Delta f_{B}$ to measure the local Brillouin spectrum and fit the spectrum with a Lorentzian function. With additional delay by data transmission and data processing, this results in a measuring time of 14 h. This measuring time could be significantly reduced by the slope-assisted approach [13,14]. Nevertheless, our BOFDA method could only be used for static changes of temperature and strain. During the measurement, a long-term stability for all components, especially lasers and modulators, should be ensured.

We also investigated a VNA with a small IF bandwidth of 50 Hz and 25 averages and compared this with one with an IF bandwidth of 500 Hz and 250 averages, and both required the same measuring time. The SNR was improved using the setting of 500 Hz and 250 averages.

**Figure 2:**
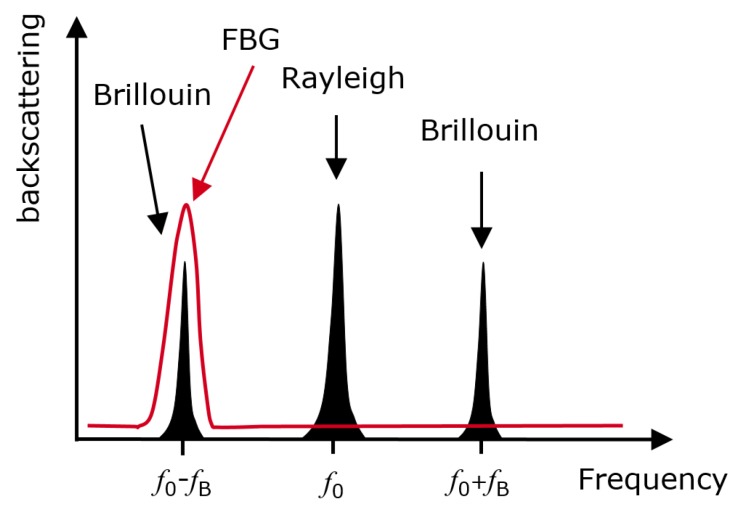
Black: Schematic of the frequency spectrum components without filtering, red curve: Filtering by the FBG of Rayleigh scattering and the upper sideband generated by the MZM1 of the probe.

The pump power modulated by the VNA was also attenuated and sent into a 3-port circulator, which passes the signal to the optical fiber and afterward receives the scattering signal, which is amplified by the probe signal. The Rayleigh scattering peak and the upper sideband generated by the MZM1 of the probe (Figure 2, red curve) were filtered out by a narrow-band fiber Bragg grating (FBG). This FBG has a full-width-half-maximum (FWHM) of 16 pm (2 GHz), adjustable around 1550 nm. The last steps of the measurement procedure necessary for sensing consisted of the opto-electrical conversion by a narrow band photo-detector (DC-35 MHz) and the measurement of the complex transfer function by a VNA.

The key point of BOFDA is the measurement of the complex transfer function. The iFFT of the complex transfer function leads to the impulse response of the sensing fiber, which is directly measured in the case of BOTDA. From system theory, it is known that the Fourier transformation of the transfer function results in the impulse response, if one assumes a linear and time-invariant system [21]. To fulfill the first requirement of linearity, a low pump and probe power of 320μW and 22μW was set in order to avoid nonlinear Brillouin interaction [21]. Additionally, the other system components, such as the photo diode and the modulators, have to operate in the linear region. The linear operation of the pump modulator depends on the modulation index $m_{p}$, which was thus chosen to be 0.5 [3]. The second requirement of time invariance requests for static temperature and strain within one measurement. This means it is impossible to detect fast changes of temperature and strain.

**Figure 3:**
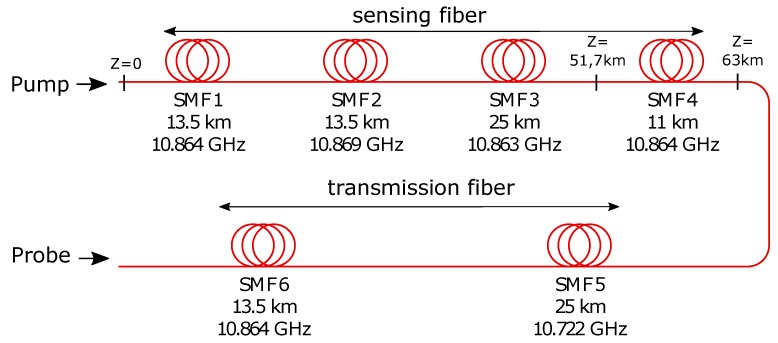
Structure of the fiber loop. Information about length and $\Delta f_{B}$.

Similar to [15], optical fibers with different BFSs were used. Our fiber loop consists of sensing and transmission parts (Figure 3). The sensing part was realized by connecting four fibers of nearly the same BFS. Fibers 3 and 4 have nearly the same BFS for a smooth transition, and a better contrast is reached for the hotspot in between the fibers. Fiber 5 has a different BFS of 10.722 GHz, and Fiber 6 has nearly the same BFS of the fibers in the sensing part.

While the two fiber ends at 52 km were spliced, the other fiber ends were connected with APC connectors. To better understand this configuration of fibers with different BFSs, the following point has to be considered: The use of a high pump power led to pump depletion [21], which reduces the sensing range. For that, we used a low pump and probe power of 320μW and 22μW, respectively. The longer the sensing fiber, the lower the power to be used. Large BFS differences among these fiber parts ensure that the Brillouin interaction can occur within only the sensing fiber, which reduces our effective sensing length and enables the use of a higher pump power [15].

## 3. Experimental Results and Discussion

**Figure 4:**
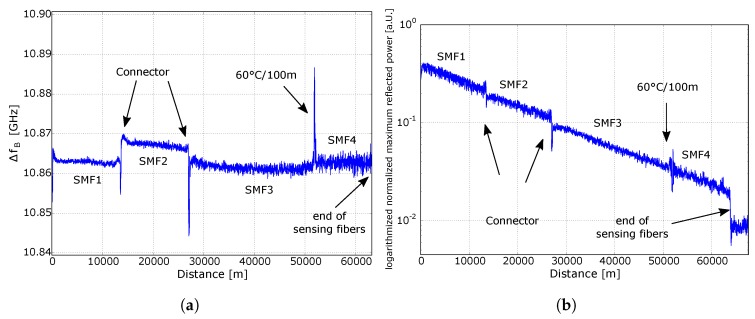
(**a**) BFS over distance (**b**) Logarithmized normalized maximum reflected power of the Brillouin spectrum over distance.

**Figure 5:**
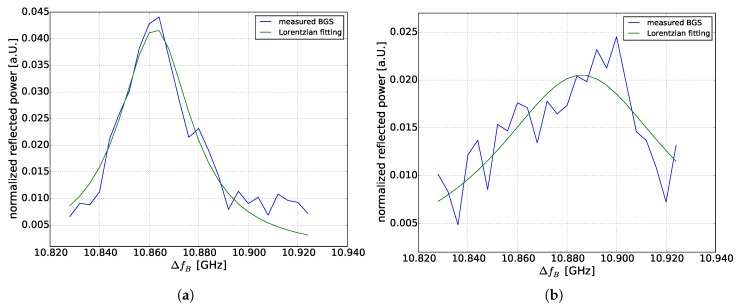
(**a**) BGS at 50 km at room temperature (**b**) BGS at 51.7 km within the heated section.

Figure 4a shows the Brillouin frequency shift $\Delta f_{B}$ over distance of the sensing part of the optical fiber. The frequency dips at 13 and 27 km, respectively, occurred due to losses at fiber connectors. Subsequently, the SNR decreases and a randomly shifted BFS was calculated by a Lorentzian fit. At 52 km, a 100 m long segment of the fiber was placed in a temperature chamber at 60 ${}^{\circ }$C. Room temperature was 23 ${}^{\circ }$C. The BFS of the 100 m long hotspot section increased by 25 MHz. This corresponds to a temperature coefficient of 0.68 MHz/${}^{\circ }$C. This coefficient is slightly lower compared to usually reported Brillouin temperature coefficients [5]. The reason for that is the effect shown in Figure 5b and is explained below. The measurement uncertainty of the BFS in the first two sections in Figure 3 (corresponding to “SMF1” and “SMF2”) was approximately 2 MHz, in the third section (“SMF3”) approximately 4 MHz, and in the last section (“SMF4”) approximately 6 MHz. By using a temperature coefficient of 0.68 MHz/${}^{\circ }$C, this corresponds to a temperature uncertainty of 2.9, 5.8, and 8.7 ${}^{\circ }$C, respectively. With increasing fiber length, the signal power decreases, which reduces the SNR and increases the uncertainty.

Figure 4b shows the amplitude level of the reflected and amplified pump power within the first 63 km of the fiber. At 63 km, a clear drop to noise level is visible, which is the end of the sensing fiber and the start of the transmission fiber. At that distance, the fiber with a different BFS at 10.722 GHz begins. The BGS of the first 63 km was measured in a frequency range from 10.828 to 10.924 GHz. A high noise of the first 27 km is observable, which is probably due to an non-uniform fiber drawing process or to a non-uniform winding process and not to the noise of our setup.

Figure 5a shows a BGS at room temperature. Figure 5b shows a BGS at 51.7 km within the heated section. There is a discrepancy between the maxima of the Lorenztian fit and the measured BGS. In fact, we would expect only a maximum at 10.9 GHz (a BFS of a 37 ${}^{\circ }$C temperature event, 60 ${}^{\circ }$C in the temperature chamber, and a 23 ${}^{\circ }$C room temperature). However, we also measured a real signal at 10.86 GHz and not noise. This effect is well known for BOFDA [25,26]. The unexpected maximum at 10.86 GHz is called a “ghost” peak. The explanation of the authors are distorting terms caused by the stationary component of the pump and the alternating component of the acoustic wave. These terms describe the prolonged backscatter of the pump wave produced by a decaying acoustic wave, even in the absence of electrostrictive force [26]. Due to this effect, our fitting routine determined a maximum of BGS at 10.88 and not 10.90 GHz. To surpress this “ghost” peak, a digital high pass filtering of the transfer function [27] will be added.

**Figure 6:**
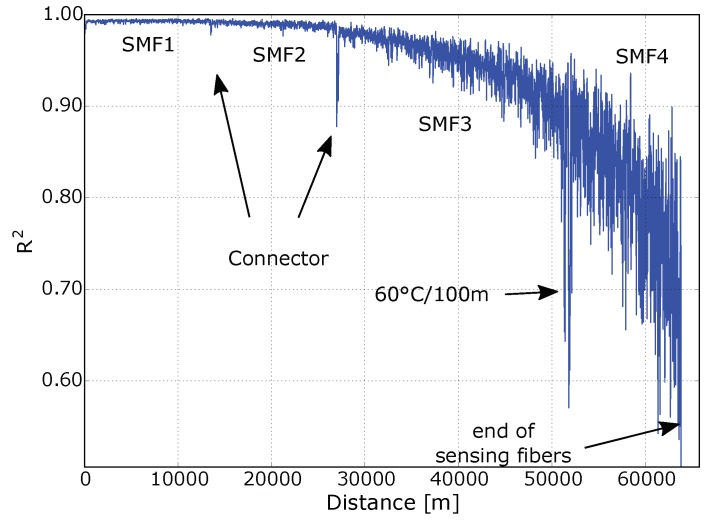
$R^{2}$ of fitting with Lorentzian function over distance.

Figure 6 shows the coefficient of determination $R^{2}$ of the Lorentzian fit. For distances above 50 km, the SBS gain approaches the noise level. The fitting error increases significantly and the fitting confidence coefficient decreases accordingly.

**Figure 7:**
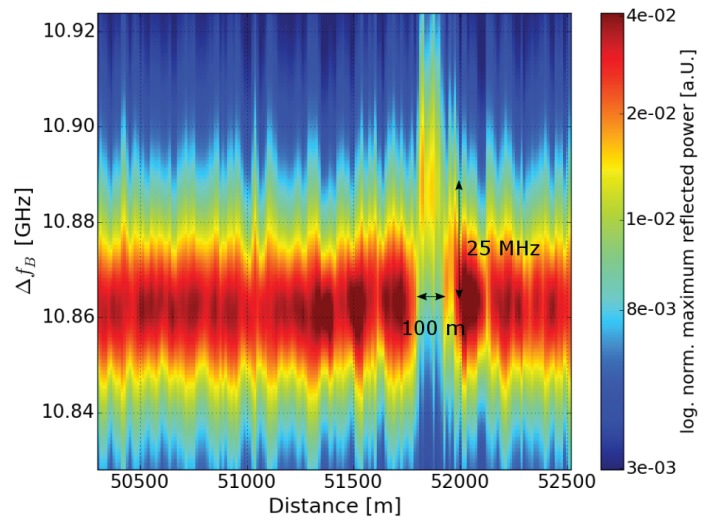
Color plot of logarithmized normalized reflected power of a 100 m fiber section at 51.7 km in a temperature chamber at 60 ${}^{\circ }$C.

In Figure 7, we show a section of the fiber loop close to the position of the hotspot. The normalized reflected power is depicted in log-scale for a better contrast of colors. At 51.7 km, 100 m of the fiber was placed in a temperature chamber. This signal was measured with a spatial resolution of 26 m. The 100 m hotspot could be clearly detected.

In future, a distributed Raman amplification [7,9,28] will be implemented in order to extend the measurement range. Moreover, for the same measurement length, the Raman amplification will increase the SNR. This higher SNR could be used to reduce measurement time significantly by reducing the number of averages. The above-mentioned filtering of the complex transfer function will be added to the signal processing to suppress the distortion of the Brillouin peak. Moreover, a distributed strain measurement will be investigated.

## 4. Conclusions

We are the first to demonstrate a 63 km BOFDA for temperature and strain monitoring using a 100 km fiber loop, with an accuracy of 5.8 ${}^{\circ }$C and a spatial resolution of 26 m. Because of the relatively long measurement time, our BOFDA sensor technique is suitable for static, slow-changing temperature and strain, as in the case of long-term monitoring of geotechnical structures and subsea cables. This paper presents results achieved using a basic BOFDA sensor technique without distributed Raman amplification or image processing.

## Abbreviations

The following abbreviations are used in this manuscript:

BOFDA	Brillouin Optical Frequency-Domain Analysis
BOTDR	Brillouin Optical Time-Domain Reflectometry
BOTDA	Brillouin Optical Time-Domain Analysis
CW	continuous wave
SNR	signal-to-noise-ratio
SSMF	standard single mode fiber
BFS	Brillouin frequency shifts
LD	laser diode
DFB	distributed feedback
MZM	Mach–Zehnder modulator
SBS	stimulated Brillouin scattering
DSB-SC	dual sideband–suppressed carrier
VNA	vector network analyzer
BGS	Brillouin gain spectrum
EDFA	erbium-doped fiber amplifier
VOA	variable optical attenuator
PS	polarization scrambler
iFFT	inverse Fast Fourier Transformation
IF	Intermediate Frequency
FBG	fiber Bragg grating
FWHM	full width at half maximum
APC	angled physical contact
